# Systems and Molecular Biology of Longevity and Preventive Medicine: Brain-Energy–Microbiome–Exposome Synergies in Blue Zones and the Cilento Case

**DOI:** 10.3390/ijms26167887

**Published:** 2025-08-15

**Authors:** Silvana Mirella Aliberti, Mario Capunzo, Richard H. W. Funk

**Affiliations:** 1 Department of Medicine, Surgery and Dentistry “Scuola Medica Salernitana”, University of Salerno, Baronissi, 84081 Salerno, Italy; mcapunzo@unisa.it; 2 Complex Operational Unit Health Hygiene, University Hospital San Giovanni dii Dio e Ruggi d’Aragona, 84131 Salerno, Italy; 3 Division of Preventive Medicine, Dresden International University (DIU), 01067 Dresden, Germany; richard.funk@tu-dresden.de; 4 Institute of Anatomy, Technische Universität (TU) Dresden, 01307 Dresden, Germany

**Keywords:** longevity, healthspan, autonomic nervous system, energy metabolism, gut–brain axis, telomeres, epigenetics, exposome, Cilento, Blue Zones

## Abstract

Longevity and healthy aging result from the complex interaction of genetic, epigenetic, microbial, behavioral, and environmental factors. The central nervous system—particularly the cerebral cortex—and the autonomic nervous system (ANS) play key roles in integrating external and internal signals, shaping energy metabolism, immune tone, and emotional regulation. This narrative review examines how the brain–ANS axis interacts with epigenetic regulation, telomere dynamics, the gut microbiome, and the exposome to influence biological aging and resilience. Relevant literature published between 2010 and 2025 was selected through comprehensive database searches (PubMed, Scopus, Google Scholar), with a focus on studies addressing the multisystemic determinants of aging. Emphasis is placed on lifestyle-related exposures, such as diet, physical activity, psychosocial support, and environmental quality, that modulate systemic physiology through neurovisceral pathways. Drawing on empirical findings from classical Blue Zones and recent observational research in the Cilento region of southern Italy, this review highlights how context-specific factors—such as clean air, mineral-rich water, Mediterranean dietary patterns, and strong social cohesion—may foster bioelectric, metabolic, and neuroimmune homeostasis. By integrating data from neuroscience, systems biology, and environmental epidemiology, the review proposes a comprehensive model for understanding healthy longevity and supports the development of personalized, context-sensitive strategies in geroscience and preventive medicine.

## 1. Introduction

Aging is a complex, dynamic, and multifactorial biological process shaped by the intricate interplay between genetic predisposition, epigenetic mechanisms, environmental exposures, the gut microbiome, the exposome, and the regulatory functions of the brain, particularly in relation to energy metabolism and neural integration [1]. Although genetic factors contribute to approximately 20–30% of the variability in human lifespan, the remaining 70–80% is influenced by modifiable determinants such as diet, physical activity, psychosocial dynamics, environmental quality, and microbial composition. This highlights the potential for preventive strategies and public health intervention [1,2]. Understanding how these modifiable factors act on biological systems is essential for designing interventions aimed at promoting healthy longevity.

Among the emerging paradigms in aging research, the concept of Blue Zones (BZs)—geographic areas characterized by exceptional longevity and sustained healthspan—has gained substantial scientific attention. First identified by Poulain and Pes [3], and popularized by Buettner [4], classical BZs include Okinawa (Japan), Sardinia (Italy), Ikaria (Greece), Nicoya (Costa Rica), and Loma Linda (USA), with Martinique recently proposed as a new addition to this group [5]. Despite their cultural and geographic diversity, these regions share common features: regular low-intensity physical activity, predominantly plant-based diets, minimal consumption of ultra-processed foods, strong social cohesion, low chronic stress, and a pronounced sense of existential purpose, often conceptualized as “ikigai” [6].

Cilento, a Mediterranean subregion in southern Italy, has emerged as a compelling candidate for inclusion among Longevity Blue Zones (LBZs). Observational and epidemiological studies [6,7,8,9,10,11] document unusually high proportions of nonagenarians and centenarians, alongside robust indicators of healthy aging. This longevity appears to result from synergistic interactions among multiple factors: superior environmental quality (e.g., clean air and mineral-rich tap water) [7,9] traditional diet rich in polyphenols (especially from extra virgin olive oil and wild herbs), routine physical activity stimulated by the region’s terrain, and dense social networks reinforced by intergenerational bonds, religious engagement, and cultural continuity [6].

Cilento and other BZ exemplify how environmental, social, and potentially epigenetic and genetic factors, shaped across generations–interact to promote exceptional health outcomes and longevity. These regions underscore the foundational role of positive external cues, such as clean air, nutrient-rich diets, and cohesive social structures, in cultivating physiological resilience. However, the precise biological mechanisms through which these inputs are transduced into long-term systemic harmony remain a central research question.

At the core of this transduction process is the central nervous system (CNS), with a pivotal role played by the prefrontal cortex. This region integrates sensory, cognitive and social information to modulate behavior and emotional regulation, transmitting outputs via the autonomic nervous system (ANS). The ANS, comprising sympathetic and parasympathetic branches, governs visceral functions, including digestion, cardiovascular tone, and immune modulation, in close coordination with the enteric nervous system (ENS), often referred to as the “second brain”. The ENS facilitates gut–brain communication, translating dietary and microbial signals into neurophysiological outputs [12].

At the periphery, this CNS-ANS axis interfaces with immune cells, blood and lymphatic capillaries, tissue-derived hormones, and humoral mediators located within the extracellular matrix and parenchymal tissues. These components form essential hubs for systemic regulation. A further integrative dimension is represented by bioelectric signaling, characterized by the synchronization of physiological oscillators, such as electroencephalogram (EEG), electrocardiogram (ECG), respiratory rhythms, and circadian hormonal cycles [13]. The resulting state of bioelectric coherence, often facilitated in low-stress, rhythmically structured environments like those found in BZ, supports emotional regulation, reduces systemic inflammation, and enhances overall resilience.

The exposome—the cumulative suite of environmental exposures, including air and water quality, social environment, and behavioral patterns—is processed through this intricate neurophysiological network. These external stimuli are biologically interpreted and converted into internal regulatory states conducive to healthy aging [14,15,16].

To understand how such environmental and psychosocial cues are transduced into sustained physiological resilience, it is essential to examine the mediating systems that bridge external and internal domains. This review proposes an integrative systems framework—conceptualized as the brain-ANS-epigenetics-genetics-telomere-microbiome-exposome axis—as a model for investigating these interactions.

At the apex of this axis is the CNS, particularly the prefrontal cortex (PFC), which exerts top-down control over emotional and autonomic responses via connections with limbic structures [14]. The control modulates the ANS, influencing cardiovascular dynamics, immune tone, and gastrointestinal processes. The ENS further facilitates bidirectional communication between the brain and gut, with microbial metabolites playing an increasingly recognized role in this crosstalk [15].

Energy metabolism emerges as a fundamental substrate within this cascade. Mitochondrial function, glucose regulation, and cellular energy production are tightly regulated by cerebral networks, particularly the cerebral cortex, which orchestrates energy allocation to maintain homeostasis and stress resilience [17,18]. ANS signaling translates these metabolic directives into peripheral adjustments in energy expenditure, oxidative balance, and immune readiness, with downstream effects on epigenetic regulation and telomere dynamics [14].

Peripheral systems—including the extracellular matrix, immune tissues, and visceral organs—serve as active participants in maintaining systemic homeostasis. Here, autonomic nerve endings, immune cells, hormonal messengers, and bioelectric potentials interact to sustain adaptive responses to both internal and external challenges.

Bioelectrical synchronization represents a distinct layer of physiological integration. The concept of bioelectric coherence, defined as the rhythmic entrainment of neurocardiac and respiratory oscillators, is increasingly recognized as a biomarker of parasympathetic dominance and emotional self-regulation. This coherence is often heightened in individuals living within structured, low-stress socioecological systems such as BZ and Cilento, where daily rhythms, environmental exposure, and psychosocial buffering coalesce.

At cellular level, telomeres—protective chromosomal end-caps—serve as both markers and modulators of biological aging. Telomere length is sensitive to oxidative stress, psychological burden, dietary inputs, physical activity, and gut microbial composition [19]. The gut microbiome emerges as a critical intermediary, influencing neuroimmune interactions, metabolic homeostasis, and epigenetic landscapes [20].

The exposome operates as a contextual modulator of these internal systems. Airborne pollutants, waterborne elements, soil composition, and social dynamics all contribute to either resilience or vulnerability in the aging process [10,11]. For example, the mineral profile of drinking water or the presence of bioactive compounds in local vegetation may directly influence metabolic and epigenetic processes.

This narrative review aims to integrate findings from neuroscience, systems physiology, environmental and preventive medicine, behavioral sciences, and epidemiology to understand how environmental and lifestyle factors influence the biology of aging. With the background of extensive empirical data from Cilento and comparative analysis with other BZ, it seeks to:

- Clarify the biological and systemic pathways through which positive environmental and social conditions promote physiological resilience and healthy aging. internal regulatory states.
- Identify key integrative nodes–such as the CNS-ANS-ENS axis, immune-bioelectric interfaces, and the gut–brain-microbiota loop–that mediate adaptive responses across systems.
- Propose a scientifically grounded, systems-based model for aging as a modifiable process, with implications for geroscience, preventive medicine, and context-aware public health.
- Offer translational insights derived from real-world models of exceptional longevity, especially those observed in Cilento and other BZ, to guide the development of effective, evidence-based healthspan-promoting strategies.

## 2. Methods

This narrative review adopts a multidisciplinary and integrative methodology to synthesize current evidence on the interrelated roles of the central and ANS, brain-energy dynamics, gut microbiota, epigenetic regulation, telomere biology, and environmental exposures (collectively referred to as the exposome) in modulating human longevity and healthspan. The analysis employs a systems-level framework to explore how biological, behavioral, and environmental domains converge to influence aging trajectories. Methodological choices followed the SANRA (scale for Assessment of Narrative Review Articles) framework to ensure transparency, rigor, and reproducibility in narrative synthesis.

### 2.1. Literature Search Strategy

The temporal scope includes peer-reviewed literature published between January 2010 and January 2025. Three principal databases–PubMed, Scopus, and Google Scholar–were systematically queried. PubMed and Scopus were chosen for their coverage of biomedical and life sciences, while Google Scholar was additionally used to identify gray literature and recently published articles not yet indexed.

Boolean logic was applied to combine search terms, which included:

“cerebral cortex energy metabolism”, “autonomic nervous system epigenetics”, “telomere length longevity”, “gut microbiome aging”, “exposome longevity”, “lifestyle interventions aging”, “Blue Zones”, “Mediterranean diet microbiome” and “physical activity well-being”.

Medical Subject Headings (MeSH) terms were used where applicable to improve retrieval specificity.

### 2.2. Study Selection Criteria

Two reviewers (SMA and RHWF) independently screened titles, abstracts, and full texts. Disagreements were resolved by discussion and consensus.

Inclusion criteria:

- Original research, systematic reviews, or meta-analytical involving human or animal models directly addressing mechanisms of aging, resilience, and multisystemic integration.
- Longitudinal studies, Randomized controlled trials (RCTs), and observational research providing mechanistic insights into interactions among neurophysiological, epigenetic, immunological, microbial, and environmental factors.
- Studies examining emotional regulation, stress reactivity, empathy, or contemplative practices as mediators of neuroaffective and autonomic coherence.
- Comparative research on sociobiological determinants of longevity in BZ and Mediterranean populations.

Exclusion criteria:

- Studies lacking methodological rigor or transparency.
- Reports focused exclusively on pathological conditions without reference to healthy aging or resilience mechanisms.
- Publications not available in English.

### 2.3. Data Extraction and Thematic Organization

Given the narrative scope, no standardized extraction sheet was used. Instead, full-text articles were read in detail, and relevant data were manually annotated in structured notes. For each study, we recorded:

- Study design, setting, and sample characteristics.
- Primary biological or environmental variables examined.
- Main findings and reported limitations.
- Potential biases or confounding factors noted by the authors.

Extracted data were then organized thematically and mapped onto the manuscript’s main sections:

- Brain, nutriregulation, and energy metabolism.
- ANS and energy homeostasis.
- Gut microbiome.
- Epigenetic and genetic regulation.
- Telomere biology.
- Exposome.
- Insights from BZ (Cilento model).
- Physical activity and quality of life (QoL).

### 2.4. Integration of Original Fieldwork

In parallel with externally sourced literature, this review integrates original fieldwork and community-based research entirely designed and conducted by the lead author (SMA principal investigator) in the Cilento region, a proposed emerging Blue Zone. These contributions include:

o Cross-sectional analyses of extreme longevity and environmental determinants [7,9].
o Qualitative and quantitative assessments of community well-being via physician interviews and ethnographic observations [8].
o Comparative analyses between Cilento and established BZ [6].
o Investigations on the exposome, including environmental quality, dietary patterns, and social determinants, in relation to aging processes across BZ populations [10,11].

### 2.5. Theoretical Frameworks

Complementing this empirical evidence, theoretical perspectives developed by RHWF on brain-energy dynamics, consciousness, and systems biology provide a conceptual foundation for the integrative perspective on longevity and resilience [21,22,23,24].

### 2.6. Bias Mitigation

To reduce selection bias, literature searches were conducted across multiple databases and supplemented by backward and forward citation tracking. Both reviewers independently assessed studies for inclusion, resolving disagreements by consensus, to minimize subjective bias. Single-study findings were always interpreted within the broader evidence base, and potential methodological weaknesses–such as small sample sizes or cross-sectional designs, or lack of control for confounders–were explicitly acknowledged in the synthesis.

### 2.7. Data Synthesis

Evidence was integrated qualitatively, drawing on neuroscience, systems biology, environmental epidemiology, behavioral science, and public health. A thematic integration strategy was applied, grouping findings according to biological pathways and cross-domain interactions. When available, covering evidence from different methodological approaches was used to strengthen interpretative validity.

Priority was given to human studies with translational relevance, though selected preclinical models were included where necessary to elucidate mechanisms not directly observable in humans.

This integrative and observational methodological approach aims to provide a transparent, reproducible, and multidisciplinary synthesis to inform future directions in geroscience, preventive medicine, and environmental health promotion.

## 3. The Brain, Nutriregulation, and Energy Metabolism in Lifestyle-Mediated Longevity

### 3.1. Prefrontal Cortex and Lifestyle Regulation

The human brain, particular the cerebral cortex, serves as the central integrative hub that governs cognition, emotional regulation, and behavior, all of which are essential for initiating and maintaining health-promoting lifestyle choices. Among cortical regions, the PFC plays a pivotal role in executive function, including decision-making, impulse control, and long-term planning, and self-regulation [25]. It exerts top-down modulation over subcortical regions such as the limbic system and hypothalamus, thereby influencing autonomic, endocrine, and behavioral responses to both internal and external stimuli.

In addition to its regulatory functions, the brain is an energetically demanding organ, accounting for approximately 20% of the body’s total energy consumption despite comprising only 2% of body mass [26]. This high metabolic demand renders it particularly vulnerable to mitochondrial dysfunction and oxidative stress—both of which are implicated in aging process. Mitochondrial oxidative phosphorylation, while essential for Adenosine Triphosphate (ATP) production, also generates reactive oxygen species (ROS), which, if not adequately neutralized by antioxidant defenses, can damage DNA proteins, and lipids [27].

Lifestyle behaviors regulated through PFC networks, such as caloric moderation, regular physical activity, and adherence to anti-inflammatory dietary patterns (e.g., Mediterranean diet), have been shown to enhance mitochondrial efficiency, reduce ROS production, and support energy homeostasis and longevity [28,29]. Functional neuroimaging studies reveal that individuals with greater activation of the PFC are more likely to adhere to healthy lifestyle patterns, which are associated with reduced systemic inflammation, improved metabolic profiles, enhanced cognitive resilience, and psychological well-being [29].

This relationship is evident in long-living populations such as those in the Cilento region of Southern Italy. Studies [8,10,11] conducted in this area highlighted that strong adherence to a Mediterranean-style diet—rich in polyphenols, omega-3 fatty acids, fiber, and fermented foods—was associated with a range of beneficial biomarkers: longer leukocyte telomere length (LTL), reduced levels of C-reactive protein, increased mitochondrial DNA copy number, improved gut microbial diversity, and higher self-reported energy levels [30]. Dietary metabolites such as short-chain fatty acids (SCAFs), particularly butyrate, have been shown to exert both mitochondrial and epigenetic effects, further reinforcing the centrality of nutriregulation in neuroenergetic longevity [31].

### 3.2. Emotional Processing and Autonomic Modulation

Beyond cognition and metabolism, the cerebral cortex, particularly the PFC, functions as a primary hub for integrating external sensory inputs and internal interoceptive signals, translating them into emotional, behavioral, and physiological responses that profoundly influence the trajectory of aging and longevity. The PFC governs complex processes such as affect regulation, attention allocation, moral reasoning, and long-term planning, all of which contribute to lifestyle behaviors critical for healthspan.

Positive emotions, such as happiness, contentment, gratitude, and awe, are recurrent features of high-longevity populations like those in BZ. These emotions are grounded in social cohesion, regular exposure to natural environments, and culturally embedded life purpose. Neuroimaging studies have shown that such positive states predominantly activate the left PFC, while negative emotions, such as fear, stress, or anger, are more strongly associated with right PFC activity [32,33]. This emotional lateralization corresponds with broader autonomic outputs: the left hemisphere is more aligned with parasympathetic activity, while the right hemisphere facilitates sympathetic responses, a functional asymmetry substantiated by studies like Wittling et al. [34] and Schiffer [35]. This neural organization enables a fine-tuned modulation of the ANS, with downstream effects on metabolic, cardiovascular, and immune functions.

**Figure 1:**
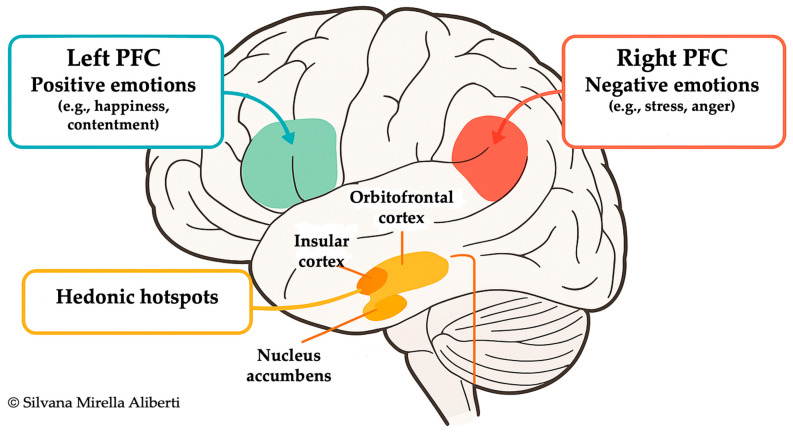
Schematic representation of brain regions involved in processing positive and negative emotions. The left PFC is associated with positive emotions (e.g., happiness, contentment), while the right PFC is linked to negative emotions (e.g., stress, anger). Subcortical regions, including the orbitofrontal cortex, insular cortex, and nucleus accumbens, contain hedonic “hotspots” that enhance emotional responses to social and environmental stimuli, prevalent in BZs like Cilento.

Beyond cortical laterality, subcortical structures exert a central influence on the regulation of hedonic tone. Specific microregions within the orbitofrontal cortex (OFC) and insular cortex operate as “hedonic hotspots”, where opioid and orexin signaling amplify ‘linking’ responses to intrinsically rewarding stimuli, including palatable food and social interactions [36,37]. These hotspots mirror functional loci in the nucleus accumbens and ventral pallidum, suggesting a distributed hedonic processing network (See Figure 1 for a schematic representation of emotion-related brain regions).

Functional neuroimaging has revealed that the mid-anterior OFC specifically encodes subjective pleasure and emotional salience [38,39,40], linking exteroceptive signals with motivational drives.

The anterior insula, highly integrative region involved in interoceptive awareness and affective social cognition, contributes not only to the current emotional state but also to the anticipation and prediction of emotionally salient outcomes. According to Singer et al. [41], its subregions (dorsal vs. ventro-central; anterior vs. posterior) support distinct yet complementary functions, including empathy, viscerosensory integration, and social attunement. The anterior insula’s role in affective social cognition extends particularly to empathy, a core function of the “emotional brain” that supports social cohesion—a key factor in long-living populations. As de Vignemont and Singer [42] propose, empathic responses are shaped by appraisal processes that integrate information about emotional stimuli, their contextual meaning, and the empathizer’s relationship with the target, thereby enabling rapid and adaptive predictions of others’ needs and actions [43]. This empathic capacity is further supported by the insula’s anatomical and functional connectivity with regions such as the OFC and the anterior cingulate cortex. These connections become especially relevant when individuals consciously attend to interoceptive signals, positioning the insula as a critical hub for integrating and regulating internal and external information [44,45]. Such mechanisms promote prosocial behavior, altruism, and cooperation—hallmarks of BZ communities characterized by exceptional longevity and social integration. These regions are also functionally connected to the claustrum, a thin sheet of gray matter between the insular cortex and the basal ganglia, which is proposed to interact with the frontoparietal control network in facilitate attentional flexibility and adaptive cognitive processing [46]. Together, this cortico-subcortical architecture offers a neurobiological substrate for environmental attunement, affective resonance, and sensory pleasure, core elements of exposomic integration in long-living populations. In addition to these structural and functional networks, recent theoretical frameworks suggest that the rapid integration of sensory and interoceptive information may also be influenced by electromagnetic fields (EMFs) and local electric fields. These biophysical phenomena have been proposed as fast-acting mediators of consciousness and large-scale information processing [22,23,47]. McFadden’s conscious electromagnetic information field (CEMI) theory posits that consciousness may emerge from spatially organized patterns of information encoded within the brain’s EM field, effectively allowing the cortex to function as an “information mixing machine” that dynamically integrates internal and external signals [48,49,50,51]. Furthermore, some emerging models suggest that quantum-level computations may contribute to the generation of subjective awareness, though such claims remain highly speculative and empirically underdetermined [52,53]. The potential interaction between electromagnetic and quantum processes may enhance the brain’s capacity for environmental attunement and affective resonance—features that may underpin emotional regulation and neurocognitive adaptability in long-living individuals.

### 3.3. Stress, Sociocultural Context, and Neuroendocrine Aging

Further affective modulation is mediated at the brainstem level. The parabrachial nucleus in the dorsal pons, traditionally associated with visceral reflexes, is now recognized for its role in hedonic signaling, taste integration, and emotional motivation [54]. These findings support a hierarchical model in which affective information is encoded not only cortically but also in deeper neural structures that directly influence the ANS and limbic circuitry.

Overall, positive emotional states—through the activation of this extended cortico-subcortical-brainstem network—are reliably associated with enhanced parasympathetic activity and vagal tone. This promotes an anabolic physiological milieu conducive to tissue repair, immune regulation, glycemic homeostasis, and mitochondrial efficiency [14]. In contrast, chronic stress—widespread in urbanized, socially fragmented environments—activates the hypothalamic–pituitary–adrenal (HPA) axis and the sympathetic branch of the ANS, resulting in elevated cortisol, metabolic disruption, gut dysbiosis, and telomere shortening, all of which contribute to accelerated biological aging [55].

Moreover, as emphasized by Shiota et al. [56], positive emotion is not a unitary construct but encompasses diverse affective states that cannot be fully captured by traditional arousal-based models. This necessitates a multidimensional understanding of affect, particularly in the context of salutogenic environments like BZ, where diverse forms of positive emotion co-occur with biological markers of slowed aging and enhanced resilience.

It has also been hypothesized that emotional resonance and systemic coherence are supported by biorhythmic coupling mechanisms, including cardiorespiratory synchronization and neural entrainment. According to Jerath et al., the integration of cortical, brainstem, and autonomic rhythms may underlie the alignment of emotional tone, respiratory patterns, and heart-rate variability (HRV), creating a dynamic biophysical interface for psychosomatic coherence and adaptation.

These phenomena are particularly evident in BZ such as Cilento, where low-stress environments are associated with parasympathetic predominance and vagal tone elevation, supporting an anabolic physiological state favorable to repair, immunity, and metabolic stability [14]. By contrast, chronic exposure to psychosocial stressors promotes sympathetic dominance, with downstream effects on endocrine, immune, and mitochondrial systems [55].

Beyond the cortex, the hypothalamus plays a pivotal neuroendocrine role, serving as a central interface between the brain and peripheral physiology. It regulates the HPA axis and orchestrates adaptive responses to stress. Prolonged HPA axis activation—frequent in high-allostatic load settings—drives systemic dysregulation, including elevated glucocorticoid levels, insulin resistance, intestinal dysbiosis, and immunosenescence, all hallmarks of accelerated aging [55].

In contrast to urban environments, the sociocultural and ecological conditions characteristic of rural longevity zones such as Cilento appear to promote autonomic homeostasis, through reduced perceived stress, strong social integration, and deep ecological connectedness. These factors enhanced mitochondrial efficiency, energy efficiency, and immune resilience, and overall energy regulation. Key lifestyle practices—such as routine physical activity embedded in daily tasks (e.g., walking, farming), adherence to nutrient-dense traditional diets, and robust intergenerational social support—create a neurophysiological environment conducive to long-term health and cognitive vitality [6,15]. In addition, modifiable lifestyle factors such as higher education and contemplative practices may further enhance neurobiological resilience in aging populations. For instance, higher educational attainment is associated with reduced cortical thinning in advanced age, suggesting that sustained cognitive engagement mitigates neurodegenerative processes [57]. Similarly, contemplative training, such as mindfulness meditation, has been shown to promote neuroplastic adaptations by reducing stress reactivity and increasing levels of brain-derived neurotrophic factor (BDNF), thereby enhancing hippocampal volume and improving emotional regulation [58]. Collectively, these findings underscore the role of lifestyle behavioral and cognitive factors in shaping the neurocognitive scaffolding that supports healthy aging in regions like Cilento. Within this scaffolding, the thalamus—though often underrepresented in aging research—plays a central role as a sensory integrator and attentional gatekeeper, modulating cortical access to visceral, emotional, and sensory input. Its contributions to circadian rhythm regulation, salience detection, and perceptual awareness positions it as a key relay structure in the coordination of lifestyle adherence and adaptive behavior. Thus, the thalamus may represent a critical interface between environmental affordances and neurocognitive function in long-living individuals.

Taken together, this interconnected system of cortical, subcortical, brainstem, and neuroendocrine structures orchestrates a neurobehavioral matrix that converts environmental exposures—nutritional, social, sensory—into integrated physiological responses. This matrix underlies the biological embedding of lifestyle and environment and represents a critical interface for preventive geroscience, psychoneuroimmunology, and behavioral medicine.

## 4. The Autonomic Nervous System: Energy Homeostasis and Neurophysiological Mediation of Aging

### 4.1. Sympathetic-Parasympathetic Balance and Aging Mechanisms

Building upon the neurocognitive and emotional regulation mechanisms discussed above, the ANS serves as a core physiological interface mediating the impact of environmental, behavioral, and microbial signals on aging. As a bidirectional conduit between the CNS and peripheral organs, the ANS dynamically modulates cardiovascular, immune, metabolic, and gastrointestinal functions—thus acting as a core mediator of the brain–body–microbiome–exposome axis.

The sympathetic nervous system (SNS), activated in response to stress and arousal, initiates the classical “fight-or-flight” response, characterized by increased heart rate, vasoconstriction, catecholamine release, and upregulation of the hypothalamic–pituitary–adrenal (HPA) axis, resulting in elevated cortisol levels. While this acute response is adaptive, chronic activation of stress-related SNS pathways promotes a pro-inflammatory, and catabolic internal environment. Prolonged sympathetic dominance has been shown to impair immune regulation, elevate the production of ROS, and inhibit telomerase activity–the enzyme responsible for telomere maintenance–thereby accelerating cellular senescence and biological aging [55]. According to a neuroimmune-senescence integrative model, autonomic imbalance–particularly diminished vagal tone and excessive sympathetic output to lymphoid tissues such as the spleen–may lead to oxidative telomere damage, further linking autonomic function to age-related physiological decline [59]. The ANS influence on aging also extends to its direct innervation of immune tissues, where it modulates immune cell trafficking, function, and inflammatory signaling. Most peripheral tissues, including primary and secondary lymphoid organs, receive autonomic—primarily adrenergic—as well as sensory innervation. Neurochemical mediators such as adrenaline, substance P, and the neuropeptide TAFA4 regulate leukocyte mobilization, stem cell niche dynamics, and immune surveillance [60,61,62,63,64]. These neural-immune interactions form a critical interface between stress physiology and aging, implicating autonomic dysregulation as a mechanistic contributor to immunosenescence and age-related decline in tissue regeneration.

Furthermore, chronic SNS activation diverts energy from long-term maintenance processes—such as immune surveillance and tissue repair—towards immediate survival responses. This dysregulation disrupts mitochondrial function and glucose metabolism, reducing ATP availability and ultimately compromising systemic energy efficiency, a key determinant of longevity [28]. Importantly, local inflammation sites can override general autonomic control and bio-rhythmic regeneration, interrupting repair processes normally modulated by tissue hormones and immune signaling via ANS nerve ending.

### 4.2. Bioelectrical Signaling and the Neuroenergetic Interface

Recent evidence also suggests that the ANS is closely linked to peripheral bioelectrical signaling mechanisms that regulate energy regulation, intercellular communication, and cellular repair across complex biological systems. These mechanisms include slow electrical potentials and transmembrane voltage gradients that guide the behavior of epithelial, endothelial, and mesenchymal tissues, influencing processes such as regeneration, inflammation, and morphogenesis throughout the lifespan [65].

Bioelectric patterns—emerging from resting membrane potentials and propagated via gap junctions and tunneling nanotubes—are increasingly recognized as instructive signals capable of modulating epigenetic activity, telomere maintenance, and mitochondrial dynamics [66,67,68,69]. These electrical gradients work in concert with ionic flows and local electric fields to coordinate cellular states and tissue-level functions. In this context, the ANS plays a trophic role by releasing neuromodulators that act as paracrine or endocrine signals, supporting tissue adaptation, repair, and structural integrity. Notably, sustained autonomic engagement can prevent disuse atrophy and promote functional resilience [70].

Recent advances in high-resolution imaging have illuminated how non-neuronal cells utilize membrane voltages as a mode of bioelectrical communication, creating dynamic signaling networks that contribute to developmental patterning, injury recovery, and homeostatic regulation [71]. These phenomena are also synchronized with systemic physiological rhythms—such as respiration, heartbeat, and cortical oscillations—providing real-time feedback that enhances systemic coherence and stress adaptability [72,73].

Bioelectric integration is particularly evident in tissues such as muscle, vasculature, and connective tissue, where ANS input converges with local field potentials and piezoelectric responses to mechanical strain. Mitochondria, as electrosensitive organelles, respond to shifts in membrane potential and electromagnetic fields, modulating ATP production, redox balance, and nuclear gene expression. These interactions form part of the broader “biofield”, a conceptual framework describing the ensemble of endogenous electrical, magnetic, and photonic signals that are proposed to mediate the systemic coordination of physiological rhythms and adaptive responses [74]. Within this perspective, the biofield represents and integrated bioelectrical network through which the ANS may influence cellular resilience, tissue regeneration, and longevity.

Autonomic nerve terminals, particularly in the vagus and enteric systems, are anatomically positioned to interface with these peripherals signaling domains, including perineural spaces, extracellular matrix networks, and tunneling nanotubes (TNTs), which can shuttle mitochondria and microRNAs between cells [75].

Importantly, disruptions in these electrodynamic fields, whether through chronic stress, electromagnetic pollution, or loss of vagal tone–can contribute to mitochondrial fragmentation, telomere attrition, and impaired DNA repair, linking neurophysiological dysregulation to cellular aging at multiple scales [76].

### 4.3. The Gut–Brain-Immune Axis

In the gastrointestinal system, excessive SNS activity disrupts the enteric homeostasis and alters the composition of the gut microbiome. Evidence from both human and animal studies indicates that chronic stress reduces microbial richness and diversity while promoting the overgrowth of pro-inflammatory taxa such as Escherichia coli and Clostridium spp., at the ee of beneficial short-chain fatty acid (SCFA)-producing genera such as *Faecalibacterium* and *Roseburia* [77]. These dysbiotic changes can impair intestinal barrier integrity and promote systemic inflammation, contributing to broader physiological dysregulation.

The gut–brain axis, a bidirectional communication system linking the CNS and gastrointestinal tract, is critically modulated by gut-derived neurotransmitters–particularly serotonin. Approximately 90% of serotonin is synthesized in the gut, where it plays a pivotal role in autonomic signaling. Hwang and Oh [78] demonstrated that gut-derived serotonin activates vagal afferent fibers, transmitting signals to the nucleus tractus solitarius and influencing central serotonergic and noradrenergic circuits. Importantly, the gut microbiota plays an active regulatory role in serotonin metabolism and availability, thus constituting a dynamic layer in this neurochemical cascade [79].

Vagal afferents, expressing diverse serotonin receptor subtypes, transduce local gastrointestinal and microbial signals into neural outputs that regulate mood, stress responsiveness, and immune modulation. These pathways highlight the gut’s role as a neuroimmune interface. Notably, the ENS, often referred to as the “second brain”, communicates with the CNS via vagal projections to the brainstem. The ENS is deeply involved in coordinating gastrointestinal motility, chemosensation (including taste), and immune surveillance in relation to the gut microbiome. Collectively, these gut–brain interactions underscore the impact of chronic autonomic imbalance on microbiota composition, immune homeostasis, and neurocognitive function.

Energy metabolism is intimately linked to this neuroimmune-microbiota interaction: impaired mitochondrial respiration caused by SNS-driven stress leads to increased ROS production, compounding both inflammatory responses and cellular energy deficits [27]. Autonomic imbalance plays a critical role in the development of numerous age-related diseases, particularly cardiovascular and renal pathologies linked to heightened sympathetic tone [80,81].

Compounding these effects, environmental insults—components of the exposome such as heavy metals, air pollutants, and endocrine disruptors—can further dysregulate ANS balance and microbiome integrity. A cross-sectional analysis conducted in Cilento demonstrated that individuals over 90 years of age who had chronic exposure to elevated levels of lead and arsenic in drinking water exhibited shorter telomeres elevated markers of oxidative stress, and a higher prevalence of age-associated pathologies, including cardiovascular and neurodegenerative diseases [9,11].

Moreover, the CNS, long considered an immune-privileged site due to restricted leukocyte access and an attenuated response to alloantigen’s, is nonetheless susceptible to localized inflammation. Chronic stress, microbiome dysbiosis, and systemic inflammation can compromise the blood–brain barrier (BBB), permitting entry of peripheral immune cells and cytokines that contribute to neuroinflammation. This cascade is increasingly recognized as a driver of neurodegenerative processes, including Alzheimer’s disease and age-related cognitive decline [82,83]. Positive emotional stress, by contrast, may mute these inflammatory cascades: Danner et al. [84], and Fredrickson & Levenson [85] demonstrated that positive affect may buffer stress-induced physiological responses, thereby promoting longevity.

### 4.4. Vagal Tone, Positive Emotion, and Longevity

In contrast, the parasympathetic nervous system (PNS), primarily mediated by the vagus nerve, promotes a “rest-and-digest” physiological state that facilitates anabolic processes, tissue repair, immunoregulation, and gut homeostasis. PNS activation enhances vagal tone, reduces systemic inflammation (via the cholinergic anti-inflammatory pathway), increases telomerase activity, and supports microbial diversity. It also promotes mitochondrial efficiency, enhances glucose uptake, and reduces oxidative stress—mechanisms essential for maintaining energy homeostasis and slowing biological aging [28]. Furthermore, Kop et al. [33], demonstrated that positive emotional states, such as happiness, are associated with increased high-frequency heart-rate variability (HF-HRV), indicating enhanced parasympathetic activation.

Interventions such as mindfulness meditation, yoga, tai chi, and vagus nerve stimulation (VNS) have demonstrated measurable effects on aging biomarkers and cellular health. For example, a randomized controlled trial involving 96 participants undergoing 12 weeks of mindfulness-based stress reduction (MBSR) reported a 17% increase in telomerase activity, maintenance of telomere length, a 25% rise in Lactobacillus abundance, and significant improvements in both physical and psychological well-being [86,87]. These effects are mediated, in part, by the ANS, whose nerve terminals influence gene regulation through neurotransmitter signaling cascades.

Specifically, chronic stress-related signaling involving excitatory amino acids, glucocorticoids, endocannabinoids, and BDNF induces epigenetic modifications such as histone acetylation (HA) and CpG methylation/hydroxymethylation. These alterations can disrupt gene expression, reduce genomic stability, and accelerate biological aging [88,89,90]. In contrast, PNS activity—enhanced through relaxation-based practices—has been associated with favorable epigenetic profiles that promote telomere maintenance, mitochondrial function, and cellular resilience.

Vagal tone, a marker of parasympathetic activity, has also been implicated in socioemotional processing. Steenbergen et al. [91] demonstrated that transcutaneous VNS enhanced recognition of emotional body expressions, suggesting a role for the vagus in facilitating safety perception and affective resonance.

In longevity regions such as Cilento and other BZ, PNS-supportive behaviors are embedded in daily life. Regular low-intensity physical activity, strong social ties, spiritual rituals, and a low-stress lifestyle collectively contribute to parasympathetic predominance. These behavioral patterns are associated with lower incidence of chronic diseases, correlate higher prevalence of butyrate-producing gut flora, and significantly reduced cardiovascular mortality—up to 50% lower in Ikaria compared to national averages [3,6,92,93]. Moreover, a study in Cilento reported that physically active centenarians had higher mitochondrial DNA copy number and greater ATP production capacity compared to sedentary peers [6,10], suggesting a robust link between autonomic regulation, energy metabolism, and healthy longevity.

Additionally, bioelectric coherence—manifested through synchronized rhythms in ECG, EEG, breathing, and hormonal cycles—emerges as a novel integrator of systemic health. These bioelectric rhythms, often anchored by the heart’s autonomic pacing, provide a real-time feedback system for systemic stability. Centenarians in Cilento exhibit heightened vagal tone and systemic bioelectric coherence, correlating with reduced inflammation and mitochondrial efficiency [10,11]. These findings support the idea that afferent vagal input—constituting over 80% of vagus fibers—conveys subconscious information from the “belly brain” to the cortex, shaping mood and promoting homeostasis.

Finally, neuroanatomical substrates such as the meso-prefrontal dopamine pathway—which loops through the limbic system, cingulum, and ventral tegmental area—provide a structural interface for motivation, positive affect, and executive functions [75]. The nucleus accumbens, via dopamine and enkephalin release, mediates both reward and the risk of dysregulation through addiction [94]. Positive emotions—ranging from awe and compassion to satisfaction and hope—though difficult to pinpoint physiologically, are associated with broadened cognitive and behavioral flexibility and may reinforce vagal balance [95].

The ANS thus represents a critical neurophysiological conduit through which lifestyle and environment interact with molecular aging pathways. Understanding and harnessing this axis offers promising avenues for geroprotective interventions and for reshaping the foundations of preventive and personalized medicine.

## 5. The Gut Microbiome as a Central Regulator of Energy Metabolism and Longevity

Its composition and functional capacity are profoundly influenced by lifestyle factors—diet, physical activity, psychosocial stress, social connectedness, and environmental exposures—which act through the CNS and ANS via the gut–brain axis [15].

The gut–brain axis, in particular, relies on afferent and efferent vagal pathways, immune signaling, microbial metabolites, and neuroendocrine crosstalk to maintain bidirectional communication between the microbiota and central neural structure [96]. This complex circuitry enables microbiota-derived metabolites to influence mood, cognition, and hypothalamic control of appetite and energy expenditure.

Diet stands out as a primary modulator of microbial composition and energy balance. The Mediterranean diet, prevalent in BZ such as the Cilento, supports microbial diversity by providing complex polysaccharides and polyphenols that serve as substrates for beneficial bacteria, including *Bifidobacterium*, *Lactobacillus* and *Faecalibacterium prausnitzii*. These microbes’ ferment dietary SCFAs—notably butyrate, acetate, and propionate—which act as energy substrates for colonocytes, reduce systemic inflammation, enhance gut barrier integrity, and modulate gene expression through histone deacetylase (HDAC) inhibition [31].

Among these, butyrate is particularly influential: it activates peroxisome proliferator-activated receptor gamma coactivator 1-alpha (PGC-1α), a key transcriptional regulator of mitochondrial biogenesis, thereby increasing cellular energy production by 15–20% [97].

Butyrate also modulates NAD+/NADH ratios, influencing sirtuin activity (*SIRT1*), with downstream effects on DNA repair, inflammation, and mitophagy [98]. Through these pathways, SCFAs exert epigenetic control over metabolic and inflammatory genes.

Preliminary evidence from long-lived populations, such as centenarians from the Cilento region, suggests that high adherence to the Mediterranean diet may positively influence gut microbiota composition, support mitochondrial function, and reduce chronic systemic inflammation [10,11].

The gut microbiome also exerts a substantial influence on telomere dynamics and cellular energetics via modulation of oxidative stress and inflammatory tone. Dysbiosis—characterized by reduced microbial diversity and a shift toward pro-inflammatory taxa—leads to increased levels of lipopolysaccharides (LPS), which not only trigger systemic inflammation but also impair mitochondrial function and accelerate telomere attrition [87].

LPS can directly activate TLR4 signaling in immune cells and endothelial tissues, leading to NF-kB-mediated transcription of pro-inflammatory cytokines. This contributes to mitochondrial dysfunction through inhibition of complex I and increased ROS leakage, compounding energetic failure and DNA damage [99].

In contrast, a balanced, SCFA-rich microbial profile upregulates telomerase activity, reduces ROS, and supports mitochondrial efficiency. A longitudinal cohort study of 1500 older adults reported that higher microbiome diversity was associated with a 12% increase in LTL, a 20% improvement in mitochondrial function, and a significant reduction in C-reactive protein (CRP) levels over a 10-year period [92].

The gut microbiota also interacts with the central nervous system, particularly in neurodegenerative conditions where energy dysregulation is prominent. A systematic review of 15 clinical trials highlighted that supplementation with specific Lactobacillus strains—such as *L. rhamnosus* and *L. plantarum*—modulated gut–brain communication, reduced neuroinflammation, improved cognitive scores, and attenuated Alzheimer’s disease progression by 20–30%, in part by enhancing brain energy metabolism and producing neuroprotective metabolites [83].

These microbial strains also modulate tryptophan metabolism via the kynurenine pathway and serotonin biosynthesis, thereby influencing neurogenesis, emotional regulation, and stress resilience through hypothalamic and limbic circuitry [100]. Moreover, gut-derived serotonin—whose levels are shaped by microbial community composition—activates vagal afferent fibers, transmitting signals to central autonomic structures and modulating mood, stress responses, and immune function [78,79]. This bidirectional gut–brain communication reinforces the microbiome’s critical role in maintaining systemic homeostasis and supporting longevity.

Collectively, these findings underscore the therapeutic potential of microbiome-targeted interventions for preserving cognitive function, enhancing emotional resilience, and extending healthspan.

Further, the gut microbiome is tightly regulated by the ANS and environmental stressors. Chronic SNS activation—often induced by psychological or environmental stress—reduces microbial diversity, increases intestinal permeability, and exacerbates systemic inflammation through translocation of LPS into circulation [77]. Environmental toxins, such as heavy metals in drinking water, can similarly disturb microbial composition and impair bioenergetic processes. For example, in the Cilento, high arsenic exposure was associated with a 15% decline in Bifidobacterium abundance and a 10% reduction in mitochondrial function [9]. These stressors also alter mucosal immunoglobulin A production and Paneth cell defensin release, weaking epithelial resilience and enabling opportunistic pathogens to displace comments [101].

Conversely, PNS activation—through practices such as meditation, forest immersion, and strong social bonds—has been associated with restoration of microbial balance, enhanced mitochondrial efficiency, and increased biosynthesis of anti-inflammatory SCFAs, thereby promoting systemic resilience and healthy aging [102].

Such practices may also upregulate vagal afferent sensitivity, enhance gut peristalsis, and improve mucosal blood flow–factors that reinforce the ecological stability of the gut environment and maintain tight junction integrity [103].

## 6. Epigenetic and Genetic Regulation

Building upon the central role of the microbiome and mitochondrial function, epigenetic mechanisms—including DNA methylation, histone modifications, and non-coding RNA regulation—act as critical interfaces between environmental inputs and gene expression programs that govern energy metabolism, cellular stress resistance, and longevity.

These mechanisms are dynamically modulated by lifestyle factors (e.g., diet, sleep, physical activity), microbial metabolites, the exposome, and mitochondrial efficiency, ultimately regulating genes involved in inflammation, oxidative stress response, DNA repair, and mitochondrial biogenesis [28,104].

For instance, chronic psychological or physiological stress triggers hypermethylation of anti-inflammatory gene promoters such as IL-10, while a health-supportive lifestyle—including a fiber-rich diet, adequate physical activity, and restorative sleep—favors methylation patterns that upregulate antioxidant (SOD2), DNA repair (OGG1), and mitochondrial biogenesis genes such as PGC-1α. These epigenetic adaptations enhance cellular resilience and energy efficiency, counteracting biological aging [28,105]. Emerging evidence also suggests that miRNAs secreted by both host and microbes (e.g., miR-34a, miR-21) act as regulatory nodes that integrate metabolic signals and epigenetic control, influencing mitochondrial turnover, insulin sensitivity, and inflammatory tone [106].

The gut microbiome plays a central role in epigenetic modulation through the production of SCFAs, particularly butyrate. As an endogenous HDAC inhibitor, butyrate enhances HA and activates key longevity-associated genes such as *SIRT1* and *FOXO3*, which promote stress resistance, metabolic regulation, and mitochondrial efficiency [107]. In Sardinian centenarians, elevated butyrate levels were associated with hypomethylation of *SIRT1*, a 15% increase in LTL, and a 20% enhancement in mitochondrial ATP production [108].

The ANS further modulates these epigenetic effects by integrating psychosocial, metabolic, and environmental inputs. Chronic SNS activation—often induced by psychological stress—has been shown to promote hypermethylation of anti-inflammatory gene promoters and suppress expression of longevity-related genes. In contrast, PNS activity supports HA and demethylation of loci such as *SIRT1* and *FOXO3*, thereby facilitating cellular repair and resilience [109]. Stress-responsive signaling cascades involving excitatory amino acids, glucocorticoids, and endocannabinoids can induce further epigenetic modifications, including CpG methylation and histone remodeling, with consequences for genomic stability and aging trajectories [88,89,90].

Similar epigenetic signatures have been identified in Cilento centenarians, where hypomethylation of key longevity genes appears to be shaped by a distinct microbiota composition, low levels of environmental toxins, and energetically efficient lifestyles [10,11].

Genetic factors, including single-nucleotide polymorphisms (SNPs) in TERT and TERC—encoding the telomerase reverse transcriptase and RNA component, respectively—directly affect telomere maintenance [110]. The *FOXO3* gene, recurrently associated with exceptional longevity across populations, is involved in antioxidant defense, autophagy, insulin signaling, and energy homeostasis [111]. Importantly, epigenetic regulation and microbial metabolites can modulate the expression of these genes, enabling external factors to amplify or even override genetic predispositions. This interaction highlights the concept of “epigenetic buffering”, wherein lifestyle and microbial signals modulate gene expression thresholds, potentially attenuating the penetrance of deleterious alleles [112]. For example, in Okinawan centenarians, carriers of longevity-associated *FOXO3* alleles showed enhanced gene expression under a calorie-restricted, plant-based diet—a key feature of the BZ lifestyle known to optimize mitochondrial efficiency and reduce metabolic stress [113].

The ANS further integrates psychosocial inputs and physiological regulation by modulating cortisol levels, microbial ecology, and mitochondrial activity. SNS hyperactivation elevates stress hormones, alters microbiome composition, and induces pro-aging epigenetic modifications, including the repression of mitochondrial and anti-inflammatory genes. In contrast, PNS activity—enhanced through relaxation practices and social bonding—promotes histone acetylation, demethylation of anti-inflammatory loci, restoration of microbial balance, and improved mitochondrial bioenergetics [28,113]. Vagal afferents also transmit anti-inflammatory signals that suppress hypothalamic–pituitary–adrenal (HPA) axis hyperactivity, creating a feedback loop that stabilizes both the epigenome and mitochondrial function under chronic stress [103].

## 7. Telomeres, Energy Metabolism, and Longevity

As epigenetic regulation shapes gene expression in response to environmental inputs, telomeres serve as dynamic biomarkers of cellular aging and energy capacity, tightly modulated by oxidative stress, mitochondrial health, microbiome composition, and social context. Telomeres, composed of repetitive (TTAGGG)n sequences at chromosomal ends, preserve genome stability and protect against illegitimate recombination. With each cell division, telomeres progressively shorten until a critical threshold is reached, initiating cellular senescence or apoptosis—hallmarks of aging [19]. Telomere dynamics are not exclusively genetically predetermined but are modulated by a convergence of behavioral, microbial, metabolic, and environmental influences.

Oxidative stress is a principal driver of telomere attrition, fueled by chronic SNS activation, mitochondrial dysfunction, and environmental toxins. Elevated ROS damage telomeric DNA, which is guanine-rich and particularly vulnerable to oxidative lesions. Efficient energy metabolism mitigates this process: optimal mitochondrial function reduces ROS production, supports telomerase activity, and slows telomere shortening [27]. Diets rich in antioxidants (e.g., vitamins C and E, polyphenols, omega-3 fatty acids), microbial-derived SCFAs, and regular physical activity protect telomere integrity by attenuating oxidative and inflammatory insults.

The Mediterranean diet, a cornerstone of the Cilento lifestyle, exemplifies the synergistic interplay between nutrition, microbiota composition, and cellular longevity [10,114,115]. High adherence to this dietary pattern has been associated with a 15% increase in LTL compared to urban counterparts (7.2 kb vs. 6.1 kb), alongside a 15% higher mitochondrial DNA copy number, both markers of preserved bioenergetic capacity and healthy aging [116,117].

This longevity phenotype appears to be supported by the region’s high microbial diversity, including a ~40% grater abundance of *Faecalibacterium prausnitzii*, a key butyrate–producing genus. *F. prausnitzii* has been linked to reduced systemic inflammation, improved intestinal barrier integrity, enhanced mitochondrial function, and telomere maintenance, all of which contribute to increased healthspan and resilience to age-related disease [118,119].

Telomere dynamics are also shaped by psychosocial stress, which shares mechanistic pathways with pro-inflammatory immune activity and the generation of ROS, both of which accelerate telomere shortening [120,121]. Interestingly, in a Southern Italian cohort, Crocco et al. [122] reported a decline in LTL after age 70 followed by a paradoxical increase in individuals older than 92, suggesting that physically and metabolically robust individuals may experience delayed telomere attrition, potentially contributing to extended healthspan. Conversely, positive psychosocial environments and anti-inflammatory diets appear to promote telomere maintenance [123,124,125]. Physical activity, particularly when embedded in daily routines, exerts anti-inflammatory and antioxidant effects by modulating ANS balance, further supporting telomere integrity and mitochondrial function [126].

Environmental exposures also play a pivotal role in telomere biology and mitochondrial metabolism. Exposure to heavy metals such as arsenic has been linked to shorted telomeres and impaired mitochondrial function in human and animal studies [127]. These findings reinforce the importance of a clean exposomic profile in supporting longevity and cellular resilience.

Physical activity reinforces these protective mechanisms: daily walking—common in the hilly Cilento landscape—has been shown to upregulate telomerase activity by 20–30% and increase mitochondrial biogenesis by 25% [28,128]. These adaptations slow cellular aging by preserving both telomeric and mitochondrial integrity.

Finally, social engagement, a defining trait of BZ communities, has been associated with longer telomere length, improved mitochondrial function, and enhanced subjective well-being [129,130]. These benefits may reflect the impact of reduced psychosocial stress and enhanced parasympathetic activity on cellular aging and energy metabolism. Broader epidemiological data confirm these trends: robust social ties are associated with reduced SNS activity, increased PNS tone, a 20% improvement in energy metabolism, and a 25% higher abundance of butyrate-producing microbes [92,129]. In contrast, loneliness—a rising problem in urbanized societies—is linked to a 14% acceleration in telomere shortening over five years, often co-occurring with dysbiosis and elevated systemic inflammation due to increased LPS production [131].

These findings underscore the converging pathways through which energy metabolism, microbial ecology, psychosocial experience, and epigenetic regulation co-determine biological aging trajectories. As the next section will explore, these systemic interactions are mirrored in neurodegenerative and cognitive aging processes, where energetic failure, neuroinflammation, and microbiota disruption play central roles.

## 8. The Exposome and Its Role in Healthy Aging and Energy Metabolism

The exposome–defined as the cumulative measure of all environmental exposures throughout the lifespan–has emerged as a crucial determinant of biological aging, energy metabolism, and disease resilience [2]. In longevity hotspots such as Cilento, the exposome is notably favorable, marked by clean air, low levels of heavy metal contamination in water, access to mineral-rich soil, and close proximity to nature. These features, combined with nutrient-dense Mediterranean diets, daily physical activity integrated into routine tasks, and strong community ties, shape a systemic environment that promotes oxidative balance, mitochondrial function, and emotional well-being.

In this context, the health-promoting exposome observed among longevity-associated populations, such as those in the Cilento region, has been linked to higher relative abundance of *Akkermansia muciniphila*, a mucin-degrading bacterium associated with enhanced intestinal barrier function, reduced inflammation, and mitochondrial biogenesis [132]. Increased levels of *A. muciniphila* have also been associated with longer LTL and improved markers of metabolic health.

Epidemiological data suggest that exposure to airborne and waterborne pollutants correlates with reduced systemic inflammation, increased mitochondrial DNA (mtDNA) copy number, and longer telomeres, likely through the mitigation of oxidative stress [133]. For example, studies have shown that chronic exposure to heavy metals like arsenic and led is associated with decreased mtDNA content, shorter telomeres, and higher cardiovascular risk.

Moreover, the exposome plays a crucial role in shaping gut microbiota composition. Exposure to environmental toxins and air pollution has been associated with reduced microbial diversity and diminished short-chain fatty acid (SCFA) production, negatively affecting mitochondrial efficiency and immune regulation [134]. In contrast, pristine rural environments have been linked to greater microbial richness and resilience [10]. For example, rural populations adhering to traditional dietary and lifestyle patterns—such as those in Mediterranean regions like Cilento—have been reported to exhibit a higher relative abundance of beneficial taxa, including *Akkermansia muciniphila*, compared to urban dwellers [135]. These findings suggest that a clean exposomic environment may act synergistically with the microbiota–mitochondria axis to promote healthy aging.

Emerging evidence also points to the exposome’s influence on subtle bioelectric and electromagnetic processes—collectively referred to as the biofield—which integrate physiological rhythms and contribute to systemic coherence [21,23,74]. Autonomic nerve endings are known to transmit electromagnetic signals across multiple wave frequencies, mediating trophic effects that promote tissue repair, stem cell support, and immune modulation [45,136]. These bioelectric signals, observable via advanced imaging techniques, propagate through intercellular structures such as gap junctions and tunneling nanotubes, facilitating regeneration, wound healing, and cellular adaptation [66,67,69].

**Figure 2:**
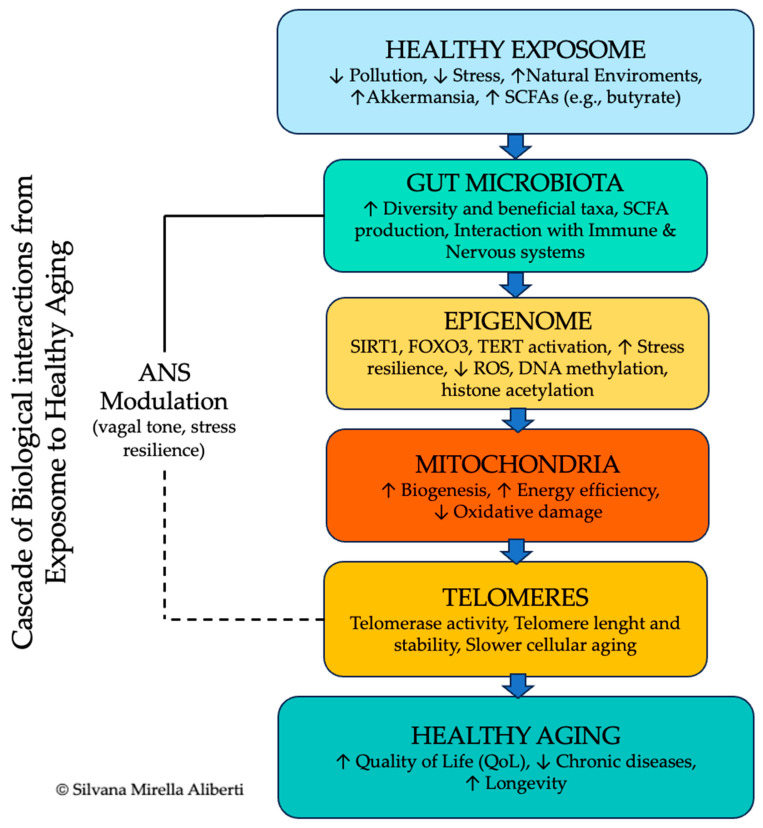
Cascade of Systems- and Molecular Biology Pathway from Exposome to Healthy Aging. This integrative model illustrates the sequential and interconnected biological pathways linking a favorable exposome to healthy aging outcomes. **Step 1—Healthy Exposome**: Low environmental pollution, reduced psychosocial stress, and rich microbial exposure increase the abundance of *Akkermansia muciniphila* and SCFAs (e.g., butyrate). **Step 2—Gut Microbiota**: Enhanced microbial diversity and metabolite production support gut barrier integrity and crosstalk with the immune and nervous systems. **Step 3—Epigenome**: Microbiome-derived and environmental signals regulate key longevity pathways (*SIRT1*, *FOXO3*, TERT), increasing stress resilience, reducing oxidative stress, and modulating epigenetic markers (DNA methylation, histone acetylation). **Step 4—Mitochondria**: Improved biogenesis and energy efficiency further lower oxidative damage. **Step 5—Telomeres**: Telomerase activity and telomere stability slow cellular aging. **Cross-cutting Modulation**: The ANS, particularly through parasympathetic vagal tone, modulates stress responses and supports homeostasis across all levels. **Outcome**: These synergistic mechanisms collectively enhance QoL, reduce chronic diseases risk, and promote longevity. Abbreviations are as listed in the manuscript’s abbreviations table; **↑** = increase; **↓** = decrease. For a detailed mapping of each pathway component and corresponding preventive/clinical strategies, see Table 1, which follows the same sequential structure illustrated in the figure.

In low-pollution, high-coherence environments such as Cilento, these bioelectromagnetic dynamics may contribute to enhanced subjective vitality and the characteristic “joie de vivre” reported in BZ populations [137,138]. However, it is important to acknowledge that while suggestive, the mechanistic underpinnings of these phenomena remain speculative. Current measurement technologies are not yet sufficiently sensitive to fully characterize or validate the functional role of biofield interactions in human physiology [74,139]. The relationships among the exposome, microbiome, epigenetics, and ANS are summarized in Figure 2.

**Table 1:** Factors linking the Exposome to Healthy Aging and corresponding Preventive/Clinical measures.

Factor	Role in Longevity	Mechanisms Involved	Examples/Zones	Preventive/Clinical Implications
**Exposome**	Influences biological aging via environmental quality and pollutant load	Environmental toxins, microbiome-mitochondria axis, endocrine disruptors, noise, light	Cilento, Nicoya, Ikaria, Martinique, Loma Linda	Air and water quality monitoring; low-pollution housing design; urban green planning; reduction in endocrine disruptor exposure in diet and household
**Microbiome**	Modulates inflammation, improves metabolism, enhances immune and cognitive resilience	SCFA production (butyrate), NAD+, AMPK, sirtuins, autophagy	Mediterranean, fermented diets, Martinique	Targeted probiotic/prebiotic supplementation; SCFA-enhancing diets (fiber-rich, polyphenols); microbiome profiling for personalized nutrition
**Epigenetics**	Tunes gene expression in response to lifestyle and environment	DNA methylation, histone modification, microRNA, HDAC inhibition via SCFAs	Plant-based, polyphenol-rich diets	Polyphenol supplementation (resveratrol, EGCG); lifestyle interventions tracking epigenetic biomarkers; nutrigenomic counseling
**Energy Metabolism**	Enhances mitochondrial efficiency, buffers stress, supports cellular homeostasis	HRV, HPA axis, vagal tone, PGC-1α, mitochondrial biogenesis	Cilento, yoga traditions, Loma Linda	Vagal nerve stimulation (non-invasive devices); regular aerobic exercise; yoga/pranayama; Mediterranean-style diet rich in mitochondrial cofactors (CoQ10, polyphenols)
**Telomeres**	Maintains genomic stability, delays cellular senescence	Telomerase activation, anti-inflammatory signaling, oxidative stress reduction	Cilento, Nicoya, Okinawa	Stress-reduction programs; antioxidant-rich diets (vitamins C, E, carotenoids); structured exercise; omega-3 fatty acids for telomere protection
**Cerebral Cortex**	Governs conscious life choices, stress resilience, and behavior regulation	Top-down control, neuroplasticity, executive function, neuroimaging	All BZ, Loma Linda	Structured cognitive training programs; MBSR; community education on health literacy; promotion of lifelong learning

Notes: Acronyms are defined in the abbreviations table at the end of the manuscript; Exposome = The cumulative measure of environmental.

## 9. Insights from Blue Zones: The Cilento Model

BZ—regions where populations achieve exceptional longevity–offer empirical insight into how synchronized regulation of the exposome, energy metabolism, and lifestyle behaviors can slow biological aging. These include Okinawa (Japan), Sardinia (Italy), Ikaria (Greece), Nicoya (Costa Rica), Loma Linda (USA), and more recently Martinique [6,11,140]. Although not officially listed, Cilento aligns with all BZ criteria, making it a compelling model for integrative longevity.

Core characteristics of the Cilento longevity model include:

- Low levels of environmental pollutants.
- Predominantly plant-based, polyphenol-rich Mediterranean diet.
- High levels of daily physical activity.
- Tight-knit social structures and intergenerational bonding.
- Optimized energy metabolism and mitochondrial efficiency [6,10].

Residents of Cilento exhibit reduced systemic inflammation–evidenced by lower circulating levels of C-reactive protein (CRP) and interleukin-6 (IL-6)–alongside enhanced mitochondrial bioenergetics and favorable epigenetic profile. Notably, hypomethylation of genes such as SIRT1 and *FOXO3*, which are critical for autophagy, stress resilience, and mitochondrial biogenesis, has been observed [113]. Abundant populations of SCFA-producing microbes, including *Faecalibacterium prausnitzii* and *Roseburia*, further support histone acetylation and activation of these longevity-associated pathways [107].

These molecular interactions are mirrored in physiological markers of healthy aging. Previous studies have shown that individuals adhering to Mediterranean-style lifestyles—often observed in rural regions like Cilento—tend to exhibit longer leukocyte telomeres and enhanced mitochondrial function compared to urban populations, likely reflecting lower oxidative stress and a more favorable exposomic profile [116,117].

The synchronized interaction of microbial, epigenetic, and metabolic factors may also engage subtle bioelectric processes—referred to as the biofield—which integrate physiological rhythms and contribute to subjective well-being, potentially underpinning the holistic vitality observed in BZ populations [137,138].

In sharp contrast, urbanized lifestyles are typified by air and noise pollution, consumption of ultra-processed foods, physical inactivity, social fragmentation, and chronic metabolic dysregulation—all of which contribute to accelerated aging and disease risk [141,142].

## 10. Physical Activity, Energy Metabolism, and Quality of Life

Physical activity represents a central modulator of the microbiota-mitochondria-brain axis, sustaining both cellular energy metabolism and systemic homeostasis. In long-lived populations such as those in Cilento and other BZ, physical activity is naturally embedded in daily life through habitual movements—walking to local markets, cultivating gardens, and navigating hilly terrain—rather than structured exercise routines. This consistent, lifelong engagement in moderate activity supports an energetic physiological tone and is associated with upregulation of telomerase activity by 20–30%, approximately 15% longer telomeres, and a 25% increase in mitochondrial biogenesis and ATP production [10,11,28,128]. At the molecular level, these effects are mediated by activation of PGC-1α, enhanced glucose uptake, improved mitochondrial dynamics, and a 25% increase in the abundance of *Akkermansia muciniphila*, which contributes to mucosal immunity and metabolic regulation [92]. Importantly, physical activity also improves psychological well-being in at-risk populations. A nationwide cross-sectional study in Italy (*n* = 1200) demonstrated that individuals engaging in at least 150 min of moderate weekly physical activity exhibited a 30% increase in perceived QoL (WHOQOL-BREF), a 25% reduction in depressive symptoms, a 20% improvement in self-reported well-being, and a 15% enhancement in mitochondrial function [93]. Moreover, physical activity attenuates the biological impact of environmental stressors. Urban dwellers who remained physically active showed 15% lower levels of inflammatory biomarkers and a 10% improvement in mitochondrial efficiency compared to sedentary peers, underscoring the protective synergy between movement and cellular energy metabolism. These benefits are further amplified by the anti-inflammatory and antioxidant effects of physical activity on ANS balance, promoting telomere preservation and resilience through reduced pro-inflammatory signaling and oxidative stress [126].

## 11. Discussion

This review elucidates the cascading influence of the brain, energy metabolism, and the ANS on longevity and well-being, mediated through epigenetics, telomere integrity, the gut microbiome, and the exposome. The cerebral cortex, as the control center for cognition and behavior, modulates lifestyle decisions that shape energy allocation, stress responses, and long-term health outcomes. This highlights the transformative potential of cognitive-behavioral interventions—such as nutritional education, stress management, environmental awareness, and microbiome support—to enhance healthy aging trajectories.

At the physiological level, energy metabolism–primarily governed by mitochondrial function and dynamically regulated by the brain-ANS interface–emerges as a central determinant of biological aging. In BZ, including the Cilento region, which exhibits hallmark longevity traits despite its unofficial status, optimized energy use supports telomere preservation, mitigates systemic inflammation, and delays age-related pathologies [10,11,28].

The PNS, particularly via vagal pathways, orchestrates key longevity mechanisms: it enhances mitochondrial efficiency, buffers epigenetic modifications induced by chronic stress, stabilizes telomere length, and promotes microbial diversity [14]. These effects are further potentiated by regular physical activity, which promotes PGC-1α expression, enhances mitochondrial biogenesis, and increases the abundance of keystone taxa such as *Akkermansia muciniphila* [92].

The gut microbiome acts as a metabolic transducer, influencing inflammation, mitochondrial health, and gene expression via SCFAs and other bioactive metabolites. Elevated SCFAs, particularly butyrate, have been shown to activate telomerase, reduce oxidative stress, and bolster cognitive and immune resilience [20]. Moreover, microbiota-derived signals influence the hypothalamic-limbic circuitry through the gut–brain axis, modulating neurogenesis, stress reactivity, and emotional regulation via vagal afferents and serotonin biosynthesis [143]. These bidirectional loops reinforce systemic homeostasis and support cognitive health.

Environmental exposures–the exposome—shape these interconnected systems [15]. Clean, low-pollution environments like those in BZ enhance mitochondrial and microbial function and preserve genomic stability. In contrast, urban environments burdened by pollution, noise, and ultraprocessed diets accelerate biological aging and energy dysregulation [10,11]. The exposome may also interact with subtle bioelectrical phenomena–voltage gradients, local field potentials, and gap junction signaling–collectively referred to as the biofield, potentially coordinating systemic coherence and support tissue regeneration. Though still speculative, such mechanisms offer a promising frontier for translational biomedicine.

Findings from Cilento centenarians exemplify this synergy, showing enhanced mitochondrial performance, longer telomeres, lower systemic inflammation, and greater microbiome diversity. Micronutrient adequacy, abundant in their traditional Mediterranean diet, supports mitochondrial function through antioxidants (vitamins C and E, selenium, zinc), B vitamins, magnesium, and other essential minerals [144]. Caloric moderation and prolonged overnight fasting–common in this lifestyle–further improve mitochondrial efficiency, reduce oxidative damage, and activate longevity-related pathways such as AMPK and mTOR [145,146]. Recent genomic analyses of Cilento inhabitants (n = 245) confirm their status as a genetic isolate enriched in variants related to extracellular matrix organization and potentially beneficial functional traits [147], while the high polyphenol content of locally consumed products, such as the PDO “Cilento Dottato” fig, provides additional antioxidant and anti-inflammatory support [148]. Together, these genetic, dietary, and lifestyle factors create a health-promoting biological milieu that may contribute to the exceptional longevity observed in this population [6,8,10].

While the integrative approach of this narrative review enables a broad, multidisciplinary synthesis, it differs from the structured protocols of systematic reviews in its search strategy and weighting of evidence, which may introduce some selection bias. The original eight-years field research conducted in the Cilento region–grounded in robust qualitative, quantitative, and environmental assessments–remains observational in design, offering robust associative insights while recognizing that complementary methodological approaches are required for definitive causal confirmation. Potential confounding factors such as genetic predispositions, selective migration, and socio-economic variables may have influenced the observed associations. However, current scientific evidence suggests that genetics factors account for approximately 30% of lifespan variance, whereas environmental and lifestyle factors contribute around 70%, underscoring the relevance of the exposomic perspective adopted here.

Importantly, this work has already incorporated comparative analyses between Cilento and recognized longevity, BZ as well as repeated geo-mapping of demographic indicators over multiple years, providing both cross-population and longitudinal perspectives. Nevertheless, future studies–particularly mechanistic, multi-regional, and intervention-based research–will be essential to further disentangle causal pathways, refine generalizability, and optimize translational applications. These considerations define the scope of applicability while reinforcing the relevance and robustness of the present findings.

Yet significant challenges remain. Access to diagnostics, including telomere length, microbiome profiling, exposome mapping, and mitochondrial function, is limited in rural areas such as Cilento. This disparity underscores the urgent need for equitable public health infrastructures. Furthermore, the interindividual variability in genetic, microbial, and metabolic responses demands the integration of personalized, exposome-aware, and energy-based interventions [20,104]. Integration of wearable sensors, AI-driven exposome analysis, and portable metabolic assays could bridge these gaps and enable individualized monitoring in real-time.

Future research should prioritize longitudinal and mechanistic studies of the brain-ANS- microbiome-epigenome-exposome network. The development of low-cost, scalable biomarkers to track biological aging, mitochondrial resilience, and environmental impact will be essential to advance the field of translational geroscience.

### Implication for Preventive Medicine

The emerging evidence supports a paradigm shift from reactive healthcare to proactive, system-based prevention targeting root biological and environmental determinants of aging. Implications include:

- Lifestyle Interventions

Regular physical activity, a Mediterranean-style diet, and stress reduction practices enhance energy metabolism, strengthen vagal tone, and modulate epigenetic aging, and inflammation.

- Environmental Policies

Urban planning to reduce air and water pollution, expand green spaces, and control chemical exposures can recalibrate the exposome for healthier metabolic profiles and longer life expectancy.

- Microbiome-Based Strategies

Prebiotic/probiotic use, along with personalized nutrition, may enhance mitochondrial function and immune balance, especially in vulnerable or metabolically compromised populations.

- Precision Diagnostics

Biomarkers such as telomere length and mitochondrial efficiency, though currently niche, may guide targeted prevention in at-risk groups, including those with rare or chronic diseases.

Importantly, the incorporation of bioelectric profiling and digital health tools may further refine preventive strategies, allowing real-time assessment of physiological coherence and early detection of dysregulation.

The Cilento model illustrates how integrative, low-cost, and culturally adapted strategies rooted in both biology and environmental stewardship can generate substantial gains in public health and sustainability.

## 12. Conclusions

The exceptional longevity observed in BZ such as Cilento reflects a complex yet reproducible interplay between environmental integrity, social cohesion, energy metabolism, and genetic resilience. These regions serve as natural laboratories for understanding the multifactorial architecture of exceptional healthspan and lifespan. Cilento centenarians offer a compelling case: they exhibit longer telomeres, improved mitochondrial function, and resilient microbiomes–all supported by an active lifestyle, a plant-based diet, and minimal environment toxicity.

These findings advocate for real-world, scalable interventions: fostering community, promoting clean environment, and supporting nutrient-dense dietary habits. Ultimately, longevity and well-being are orchestrated by neuro-metabolic-environmental axis, rooted in the brain’s control over behavior and energy use, the ANS’s homeostatic role, and the continuous feedback from the microbiome and exposome. A holistic framework that integrates neural, microbial, energetic, and environmental signaling will be essential to advancing precision geroscience. Intervening at these critical nodes offers a strategic opportunity to optimize healthspan.

Recognizing Cilento as a bona fide BZ, PFC may not only catalyze global collaboration in geroscience but also inspire scalable models for promoting personalized, preventive, and exposome-aware healthcare.

## 13. Recommendations and Policy Proposals

Grounded in the integrative neuro-metabolic framework delineated in this review—which interlinks brain regulation, energy metabolism, autonomic function, epigenetics, the gut microbiome, and the exposome—a set of actionable, evidence-based recommendations can be proposed to inform public health policy, clinical guidelines, educational reform, and future research agendas.

1. Integrate Mitochondrial Health into Public Health and Aging Policies
  To advance energy-efficient aging as a public health priority, it is essential to:
  - Incorporate validated metrics of mitochondrial function, such as VO_2_ max, lactate threshold, and circulating mitochondrial-derived peptides, into chronic disease screening and healthy aging assessments.
  - Expand access to community-based physical activity programs, especially in underserved or rural areas, to enhance mitochondrial biogenesis, maintain telomere integrity, and reduce inflammation across the lifespan.
2. Ensure Equitable Access to Biological and Environmental Diagnostics
  Disparities in access to aging-related biomarkers hinder the implementation of precision prevention. Therefore:
  - Public health system should subsidize diagnostic platforms for telomere length, mitochondrial function, gut microbiota composition, and cumulative exposome burden, prioritizing vulnerable populations.
  - Regional Longevity and Exposome Monitoring Centers should be established–modeled on paradigms observed in BZ such as Cilento–to longitudinally monitor biological aging and environmental exposures across diverse ecological contexts.
3. Institutionalize Microbiome Health in National Nutrition and Clinical Guidelines
  Given the pivotal role of gut microbiome in modulating host metabolism, immunity, and epigenetic aging:
  - National dietary recommendations should explicitly support microbiome integrity, advocating high-fiber, polyphenol-rich, and minimally processed foods, along with the regular inclusion of fermented products.
  - Clinical practice should integrate microbiome assessments–particularly for patients with metabolic, autoimmune, or rare diseases–and leverage personalized nutrition platforms based on individual microbiota profiles and functional signatures.
4. Position Environmental Health as a Foundational Pillar of Longevity Science
  Recognizing the exposome as a primary modulator of aging biology necessitates a robust environmental health infrastructure:
  - National and municipal policy should target measurable reductions in air pollutants (e.g., PM2.5, NO2), heavy metals, and endocrine-disrupting compounds.
  - Urban design initiatives should prioritize the development of biophilic environments, including green spaces and urban BZ, to mitigate chronic stress and enhance physiological resilience.
  - Regulatory oversight must expand to include the long-term health impacts of food additives, microplastics, and packaging-related xenobiotics.
5. Embed Neuro-Metabolic-Environmental Literacy in Education and Health Promotion
  To cultivate lifelong resilience, educational frameworks must address the dynamic interplay between behavior, biology, and the environment:
  - Multilevel campaigns should be implemented in schools, families, and clinical settings to increase awareness of the brain-microbiome-energy metabolism axis and its implications for physical and mental health.
  - Curricula should include experimental learning in mindful eating, movement literacy, sleep hygiene, and emotional regulation, alongside ecological awareness and sustainability practices (e.g., eco-nutrition).
6. Promote Transdisciplinary Research and Precision Prevention
  The complexity of the aging process requires convergence science approaches:
  - Dedicated funding should support longitudinal cohort studies and mechanistic trials investigating how the exposome, energy metabolism, and the gut microbiome converge to shape aging trajectories.
  - Integrated translational platforms should bridge clinical care, environmental science, genomics, and public health, fostering data interoperability and policy-relevant insights.
  - Artificial Intelligence and machine learning applications should be developed to model personalized aging trajectories based on genome-exposome-microbiome interactions, enabling early risk detection and targeted intervention.
7. Formally Recognize and Support Emerging BZ
  Regions such as Cilento offer replicable models of sustainable longevity:
  - Criteria should be developed to formally recognize emerging BZ based on convergent evidence from epidemiological, molecular, and exposomic data.
  - These regions should serve as testbeds for scalable, culturally adapted aging interventions and international collaboration in geroscience and environmental health policy.

A shift toward an exposome-informed, energy efficient, and microbiome-aware approach to preventive medicine is not merely advantageous, it is imperative. To meet the demands of 21st-century aging societies, a unified response is required across policy, clinical care, urban design, education, and research (Table 2). Only through systemic, transdisciplinary action can we generate environments and interventions that empower individuals not only to live longer, but to live with sustained health, purpose, and resilience.

## Figures and Tables

**Table 2:** Recommendations and policy proposals.

Domain	Recommendation/Policy Proposal	Target	Objective	Biological Mechanism/Rationale
**Diagnostics** **&** **Monitoring**	Public funding for telomere, mitochondrial, microbiome, and exposome biomarkers	Governments, regional health systems	Improve access to predictive tools for biological aging	Enables early detection of metabolic decline and biological age acceleration
**Education** **&** **Literacy**	Integrate education on nutrition, stress, environment, and microbiome in schools and communities	Ministries of Education and Health	Foster awareness and autonomy in preventive health	Shapes health behaviors and enhances brain-microbiome-energy literacy
**Urban Planning** **& Environment**	Promote urban greening, walkability, and air/water quality improvements	Municipalities, local governments	Recalibrate the exposome for healthier aging environments	Reduces oxidative stress, improves mitochondrial and immune function
**Food** **&** **Nutrition Policies**	Support local agriculture and Mediterranean diets in public canteens	Agricultural ministries, schools, hospitals	Encourage adoption of nutrient-dense and sustainable diets	Promotes microbial diversity, epigenetic stability, and energy efficiency
**Accessible Physical Activity**	Fund public fitness areas, walking routes, and adapted programs for older and chronically diseases	Local governments, healthcare providers	Reduce inequality in access to physical activity	Enhances mitochondrial biogenesis, telomerase activity, and vagal tone
**Precision Preventive Medicine**	Integrate microbiome, mitochondrial, telomeric, and exposome data into prevention protocols	Health systems, clinical research centers	Promote tailored interventions based on individual biology	Enables stratified interventions and personalized risk reduction
**Territorial Health Equity**	Strengthen healthcare infrastructure in rural or under-resourced areas, including emerging BZ	National and EU-level policymakers	Address health disparities and regional diagnostic gaps	Reduces geographic inequalities in healthy aging potential
**Research** **&** **Innovation**	Fund longitudinal studies on the brain-energy-environment axis and aging biomarkers	Ministries of Research, universities, EU	Validate interventions and develop scalable diagnostic tools	Supports evidence-based, cross-domain geroscience innovation

## Data Availability

PubMed/Medline, Scopus, Google Scholar.

## Abbreviations

The following abbreviations are used in this manuscript:

BZs	Blue Zones
LBZs	Longevity Blue Zones
SCFAs	Short-chain fatty acids (e.g., butyrate, acetate, propionate)
QoL	Quality of Life
HRV	Heart-Rate Variability
HPA	Hypothalamic–Pituitary–Adrenal axis
PGC-1α	Peroxisome proliferator-activated receptor gamma coactivator 1-alpha
NAD+	Nicotinamide Adenine Dinucleotide (oxidized form)
AMPK	AMP-activated protein kinase
HDAC	Histone Deacetylase
CNS	Central nervous system
ANS	Autonomic nervous system
ENS	Enteric nervous system
EEG	Electroencephalogram
ECG	Electrocardiogram
PFC	Prefrontal cortex
ATP	Adenosine Triphosphate
ROS	Reactive oxygen species
HF-HRV	High-frequency heart-rate variability
OFC	Orbitofrontal Cortex
BDNF	Brain-derived neurotrophic factor
SNS	Sympathetic nervous system
PNS	parasympathetic nervous system
TNTs	tunneling nanotubes
DNA	Deoxyribonucleic Acid
BBB	Blood–brain barrier
HA	Histone acetylation
SNPs	Single-nucleotide polymorphisms
*FOXO3*	Forkhead Box 03
*SIRT1*	Sirtuin 1
mtDNA	Mitochondrial DNA
LTL	Leukocyte telomere length
CRP	C-reactive protein
TERT	Telomerase Reverse Transcriptase
CEMI	Conscious electromagnetic information field
EMFs	Electromagnetic fields
TAFA4	Tumor necrosis factor (TNT) Alpha-induced Protein 8-Like2
VNS	Vagus nerve stimulation
MBSR	Mindfulness-based stress reduction
LPS	lipopolysaccharides
mTOR	Mechanistic target of Rapamycin
